# Preliminary Investigation of Cecal Microbiota in Experimental Broilers Reared Under the Aerosol Transmission Lameness Induction Model

**DOI:** 10.3390/ani15243641

**Published:** 2025-12-17

**Authors:** Anh Dang Trieu Do, Khawla Alharbi, Ruvindu Perera, Andi Asnayanti, Adnan Alrubaye

**Affiliations:** 1 Center of Excellence for Poultry Science, University of Arkansas, Fayetteville, AR 72701, USA; rperera@uark.edu (R.P.); aasnayan@uark.edu (A.A.); 2 Cell and Molecular Biology Program, University of Arkansas, Fayetteville, AR 72701, USA; 3 Department of Veterinary Microbiology and Preventive Medicine, Iowa State University, Ames, IA 50011, USA; kalharbi@iastate.edu; 4 Department of Poultry Science, University of Arkansas, Fayetteville, AR 72701, USA

**Keywords:** broiler, BCO lameness, microbiota, aerosol transmission model, dysbiosis

## Abstract

Bacterial chondronecrosis with osteomyelitis (BCO), and its associated lameness, is one of the most common and devastating issues the poultry industry constantly faces, both globally and domestically. Leveraging the currently accepted “leaky gut” model of pathogenesis, this study aims to evaluate the cecal community of broilers reared under the aerosol transmission BCO induction model. A trial involving 1320 Cobb 500 broilers was conducted using the same induction model for 56 d with the following treatments: (1) positive control (PC)—untreated birds on wire-flooring pens; (2) negative control (NC)—untreated birds on litter-flooring pens; (3) LOW—birds treated with probiotic *Enterococcus faecium* spray on day-of-hatch (2.0 × 10^9^ CFU/bird); and (4) HIGH—birds treated with LOW spray combined with probiotic *Bacillus amyloliquefaciens*/*Bacillus subtilis* inclusion in the diet (492.1 mg/kg). Cecal contents were collected from six birds per treatment on d14, d28, d42, and d56 of the experiment; then, DNA was extracted and sent for 16 s V3–V4 amplicon sequencing. Returned sequences were assembled and taxonomically assigned, after which diversity indices were analyzed (including alpha, beta, and abundance). No significant effect was found between all treatments and positive/negative control groups in all timepoints, but timepoints were significantly different from each other in both alpha and beta diversity indices (*p* < 0.05). Abundance analysis also showed a high Bacillota:Bacteroidota ratio (average 18.87; *p* < 0.0001) with Bacillota (Firmicutes) dominating at 95.57% on average across all treatments, followed by Bacteroidota at 5.06% and Pseudomonadota at 2.59%. These findings characterize in detail the cecal microbiome in populations of broilers reared under the novel aerosol transmission induction model, offering further insights and possibilities into studies of BCO etiology and pathology.

## Simple Summary

Broiler leg lameness from bacterial infection is a major issue affecting the poultry industry, negatively impacting production economics and animal welfare. As there is no current effective therapeutic treatment against the disease, preventive measures—including the use of probiotics/prebiotics and non-antibiotic feed additives in the animal diet—and effective, novel research methods are important tools in this effort. This experiment explores the effects of the administration of a probiotic spray, that of a probiotic feed additive, and their combination on broiler cecal microbiota over time, using an aerosol transmission lameness induction model. The experimental results provide preliminary insights into these effects and considerations for future research in the same field, ultimately contributing to sustainable poultry production.

## 1. Introduction

The poultry industry has consistently been a powerhouse in the global agriculture sector, leading in high-volume production of affordable high-quality animal protein to feed an ever-expanding global population [1,2]. Its early adaptation of the unique vertical integration production model allows for centralized operation to exert executive decisions in a rapid and efficient manner [3] compared to other livestock sectors – such as in cattle production systems – with a staggered approach to production [4]. Coupled with modern poultry production idiosyncrasies of high flock density placement, a single production complex may see “all in, all out” movement of millions of birds every 42 days on average [5]. While such management strategies are advantageous in disease control between flocks, from the complete clearing out of birds to sanitation of the barn during downtime or as part of a rapid response to bird health emergencies [6,7], common diseases—including viral and bacterial outbreaks—still often take place due to increased susceptibility from high stocking density stress during the production period [8,9], which may result in economically devastating outcomes [10,11,12]. As such, constant safeguarding of animal health, welfare, and productivity against diseases is of utmost importance for all poultry producers. However, this may be an uphill battle due to the general shift away from antibiotic and ionophore usage in livestock production out of concerns regarding resistance in both animal and human health contexts [13,14]. Preventive measures, taken either through strict biosecurity upkeep [15] or the administration of feed additives (minerals, prebiotics, probiotics, etc.), which are intended to improve bird productivity and health parameters [16,17,18], have thus become the de facto choice for most poultry producers seeking to protect their flocks.

With regard to bacterial infections within poultry species—particularly broilers—bacterial chondronecrosis with osteomyelitis (BCO) and its associated lameness has gained notoriety in recent years due to its negative economic and welfare impacts on the industry. First reported in 1972 [19], the disease has surfaced as one of the top issues facing the poultry industry in recent years, with worldwide prevalence [20,21,22,23] and industrial communications estimating approximately 3–15% animal losses depending on outbreak severity. Signified by hallmark intractable proximal and tibial head lesions resulting in bird lameness and paralysis, BCO pathogenesis is postulated to start in the gastrointestinal tract (GIT) of the broiler as a possible route [17,24], alongside the inhalation and ingestion of disseminated etiological agents. Acquired pathogenic bacteria translocate through compromised intestinal epithelial layers, eventually depositing in and infecting weight-induced leg bone microfractures as the bird ages and faces grow-out stress [24]. In the past several years, BCO research literature has made headway in molecular underpinnings regarding various factors that govern both bacterial pathogenicity and host factors [25,26,27], as has the investigation of different feed additives and feeding strategies on the reduction in BCO lameness incidence [28,29,30] using various induction models [31,32]. Most recently, a study focusing on an electron beam-treated multivalent BCO vaccine has shown significant promise as a potential preventive treatment against the disease [33]. Regardless, a holistic understanding of the etiology of BCO warrants more research, upon which novel treatments and preventives can be developed.

The GIT microbiome has long been regarded as one of the most influential sites in health outcomes across different species [34,35,36], including poultry [37]. Comprising various bacterial communities, both ubiquitous and unique to each host species, it has been directly linked to cognitive functions and proper development of various systems within the organism [38,39]. In broilers, the GIT microbiota (of which the broiler caecum serves as a reservoir) is essential to the functional immune system and proper uptake of nutrients [40]. Dysbiosis of the GIT microbiota has been implicated in impaired broiler performance and increased susceptibility to infections and other diseases [41]. In this context, GIT microbiota dysbiosis is evidently closely connected to postulated BCO etiology and pathogenesis in the “leaky gut” model [24,41]. Although its effectiveness in clinical lameness reduction compared to the wire-flooring model has been previously documented [31], there is currently—to our knowledge—no prior study examining the potential dysbiosis associated with BCO etiology [42] in this model. Leveraging the postulated “leaky gut” mechanism of BCO pathogenesis, we hypothesize that the cecal microbiota composition for broilers reared under high-stress conditions (wire-flooring seeder birds) is similar to that of the remaining litter-flooring populations, and there is a significant treatment effect between probiotic-treated and non-treated populations. The results from this preliminary study are potentially of high relevance to both poultry producers and the scientific community at large in the elucidation of BCO etiology and pathogenesis using a simple and effective induction model.

## 2. Materials and Methods

### 2.1. Animal Use Statement

This study was approved and conducted under the University of Arkansas Agricultural Institutional Animal Care and Use Committee (Ag-IACUC) protocol #23067.

### 2.2. Experimental Design

#### 2.2.1. Pen Design and Allocation

Birds were reared over 56 days under the aerosol transmission model of BCO induction [31]. On day of hatch, 1560 day-old Cobb 500 broiler chicks (Cobb-Vantress, Inc., Siloam Springs, AR, USA) were randomly allocated to 26 floor pens (1.5 m × 3.0 m/pen) at a stocking density of approximately 750 cm^2^/chick. On d14, bird numbers were adjusted to 50 chicks per pen to maintain a final density of 900 cm^2^/chick. Pens were arranged in two rows of 13 pens and randomly assigned to treatment groups. With the exception of the wire-flooring group, which consisted of only two pens, four pens were allocated to each treatment group (initial density of 240 chicks/treatment). Treatment allocation was assigned using a randomized block design. The housing environment was equipped with automated systems for temperature, lighting, and ventilation regulation. The photoperiod was set at 23 L:1 D (20 lux) for the entirety of the experiment. Environmental temperatures were adjusted incrementally, at 32.2 °C (days 1–3), 31 °C (days 4–6), 29 °C (days 7–10), and 26 °C (days 11–14), and then maintained at 23 °C from day 15 onward. Each pen was equipped with two feeders and a dedicated water line, which was disinfected with diluted bleach and flushed with clean water prior to bird placement.

#### 2.2.2. Treatment Design

The detailed investigated treatments in the experiment from which the birds were sampled have been described in detail in our previous publication [43]. Briefly, the probiotic *Enterococcus faecium* spray solution utilized for both LOW and HIGH treatments was procured from the same commercial source (GalliPro^®^ Hatch, Novozymes, Hørsholm, Denmark) and prepared identically following manufacturer instructions for a 5× dosage (2 × 10^9^ CFU/bird) as a baseline concentration for investigation [43,44]. Boxes of 60 chicks each were uniformly sprayed once on day of hatch using an in-house manual static spraying system, with food-grade blue dye added to aid in the visualization of spray distribution uniformity.

Similarly, the triple-strain *Bacillus*-based probiotic came from a commercially available source (GalliPro^®^ Fit, Novozymes, Hørsholm, Denmark) and was mixed in the broiler diet in all production phases lasting 56 days (starter: d0–18, crumbles; grower: d18–42, pellets; finisher: d42–56, pellets) at an inclusion rate of 492.1 mg/kg. Feed was prepared at the University of Arkansas Poultry Feed Mill following industry standards and provided ad libitum to experimental broilers. Table 1 details the selected treatments (n = 1320 birds) from the experiment for further cecal community analyses.

### 2.3. Lameness Incidence

Starting from d22 of age, broiler lameness was assessed daily by prompting birds to move short distances. Birds that exhibited signs of clinical lameness, including limping gait and reluctance or inability to move, were diagnosed and recorded as clinically lame, euthanized via CO_2_ inhalation, and necropsied to determine the presence of femoral and tibial head lesions using the severity scale detailed in Alharbi et al. [43].

### 2.4. Cecal Content Collection

On d14, d28, d42, and d56 of each experiment, six (6) clinically healthy birds per selected treatment were randomly chosen and euthanized via CO_2_ inhalation. The cecal contents from each bird were aseptically squeezed into sterile 5 mL screw-cap collection tubes, immediately stored on dry ice, and subsequently stored at −80 °C until extraction took place. DNA extraction of cecal contents was conducted using the QIAGEN^®^ QIAamp PowerFecal Pro DNA kit (Qiagen, Hilden, Germany) per the manufacturer’s protocol. The preliminary concentration and quality of extracted DNA for each sample were determined via spectrophotometry (DeNovix DS-11; DeNovix, Wilmington, DE, USA) and sent to the University of Texas Southwestern Medical Center (UTSW, Dallas, TX, USA) Microbiome Core sequencing laboratory for 16S V3–V4 amplicon sequencing.

### 2.5. 16S V3–V4 Sequencing of Cecal Content Samples

High-complexity DNA was used for 16S v3–v4 amplicon sequencing. The DNA concentration was measured using the Picogreen method (Invitrogen Quant-iT™ Picogreen dsDNA Assay Kit; Thermo Fisher Scientific Inc., Waltham, MA, USA) on PerkinElmer 2030 Multilabel Reader Victor X3 (PerkinElmer Inc., Shelton, CT, USA), and the DNA integrity number (DIN) was determined on 4150 Tapestation from Agilent (Agilent, Santa Clara, CA, USA) using Agilent’s gDNA Screen Tape and Agilent’s gDNA Reagents. 16S library preparation was performed using Zymo Research’s Quick-16S™ Plus NGS Library Prep Kit (V3–V4) (Zymo Research, Irvine, CA, USA) with the following primers, per the manufacturer’s specification: 341f (mixture of 5′-CCTACGGGDGGCWGCAG-3′ and 5′-CCTAYGGGGYGCWGCAG-3′) and 806r (5′-GACTACNVGGGTMTCTAATCC-3′). The quality and quantity of each sequencing library were assessed using Agilent’s 4150 Tapestation on gDNA Screen Tape. Prior to sequencing, each library or pool was quantified using KAPA Biosystems Library Quant Kit (Illumina) ROX Low qPCR Mix (Kapa Biosystems, Wilmington, MA, USA) on an Applied Biosystems 7500 Fast Real-Time PCR system (Thermo Fisher Inc., Waltham, MA, USA). 16S libraries were sequenced on Illumina MiSeq Sequencer (Illumina, San Diego, CA, USA) using PE-250 cycle and PE-300 cycle flow cells. Raw FASTQ files were demultiplexed based on unique barcodes and assessed for quality. Nuclease-free water was used as the negative control, while ZymoBIOMICS Microbial Community Standard was used as the positive control. This microbial standard is a well-defined, accurately characterized mock community consisting of Gram-negative and Gram-positive bacteria and yeasts.

### 2.6. Data Analysis

#### 2.6.1. Cumulative Lameness Incidence Analysis

The analysis methodology we used for cumulative lameness incidence was previously published by Alharbi et al. [43]. Briefly, cumulative lameness between treatment groups was compared for significant differences at the end of the study (d56) using a binomial logistic regression generalized linear model (GLM) at a threshold of *p* < 0.05.

#### 2.6.2. Cecal Community

Returned paired-end sequencing data were processed using Divisive Amplicon Denoising Algorithm 2 (DADA2) in R v4.5.0 (R Foundation for Statistical Computing, Vienna, Austria) following a similar protocol to that detailed in Do et al. [45]. Using the package dada2 v3.2.0 and its associated workflow pipeline [46], both forward and reverse reads were left-trimmed (trimLeft) at 10 nt and truncated (truncLen) at the 250th position, with a maximum expected error (maxEE) of 5 and minimum quality score (truncQ) of 2. Taxonomy was assigned to assembled and denoised reads using the Silva v138.2 reference training dataset [47], followed by the removal of chimeric sequences and further processing of assigned sequences using the phyloseq v1.46.0 package [48].

#### 2.6.3. Statistical Measures of Diversity

Measures of diversity (α, β, amplicon sequence variants abundance) and associated statistical analyses (ANOSIM, PERMANOVA, dispersion) were processed using the packages vegan v2.6–6.1 [49] and microbiome v1.24.0 [50] with Benjamini–Hochberg adjustment. For ANOSIM, permutations were set at the default value (999), while PERMANOVA permutations were set at 9999. Visualization of analyses and figure creation were performed using the package tidyverse v2.0.0 [51]. Significant differences were determined at a threshold of *p* < 0.05.

## 3. Results

### 3.1. Cumulative Lameness Incidence

**Figure 1:**
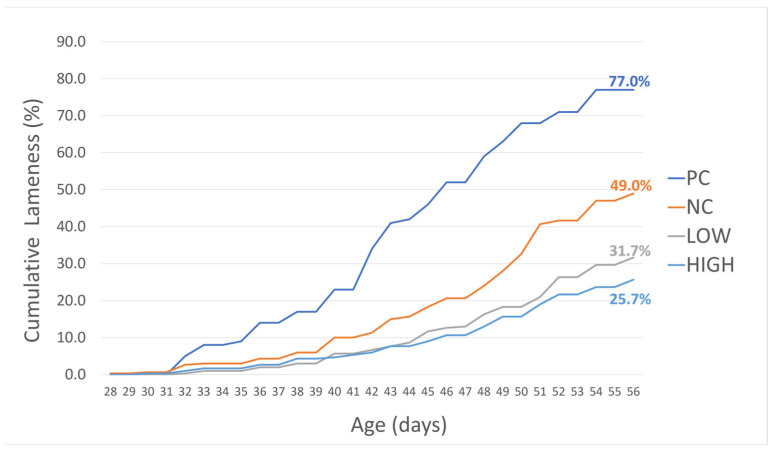
Cumulative lameness incidence rate from all experimental treatments over the course of the study. Reproduced and adapted with permission from Alharbi et al. [43].

The cumulative lameness incidence rate of all experimental treatments in this study reflected that previously published by Alharbi et al. [43]. For brevity, the results for treatments pertaining to this specific evaluation are presented below in Figure 1.

Briefly, at the end of the experiment on d56, the cumulative clinical lameness incidence for selected treatment groups was as follows: PC—77.0%; NC—49.0%; LOW (Spray only)—31.7%; and HIGH (Spray + feed combination)—25.7%. While both LOW/HIGH treatments were significantly lower in lameness incidence compared to the negative control group (Table 2), the HIGH group exhibited the greatest clinical lameness significant reduction rate of −47.6% (compared to that of the LOW group, which was −35.4%), suggesting a synergistic interaction between the inclusion of the probiotic spray and feed additive. Except for a general decrease in tibial and femoral lesion severity in LOW and HIGH groups compared to the NC group, no other significant trend was found in terms of final bird body weight and mortality between treatment groups (excluding PC) [43].

### 3.2. Alpha Diversity

**Figure 2:**
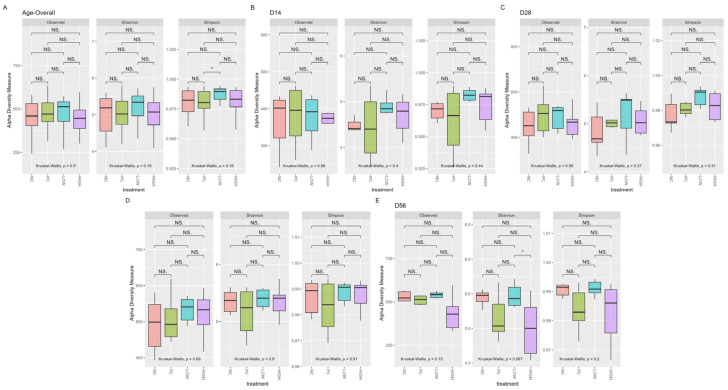
α-diversity indices (observed, Shannon, and Simpson) between treatments grouped temporally ((**A**): overall, (**B**): d14, (**C**): 28, (**D**): 42, and (**E**): 56). Significant differences within each category were determined at *p* < 0.05 based on Kruskal–Wallis test. Significant differences between each factor were determined post hoc upon pairwise Wilcoxon rank-sum comparison at *p* < 0.05 (NS. ≥ 0.05, Not Significant; * < 0.05).

Following the quality control steps described above and removal of taxa with an abundance < 0.005%, 1571 ASVs in 96 samples were retained. Overall, there was no significant difference in α-diversity indices (Observed, Shannon, Simpson) among treatments both on an overall timescale and when each collection timepoint was separately considered (Kruskal–Wallis *p* > 0.05), except for two instances in the pairwise comparison—one on an overall scale (Simpson PC vs. LOW) and on d56 (Shannon LOW vs. HIGH)—which are not considered as a posteriori results (Figure 2).

**Figure 3:**
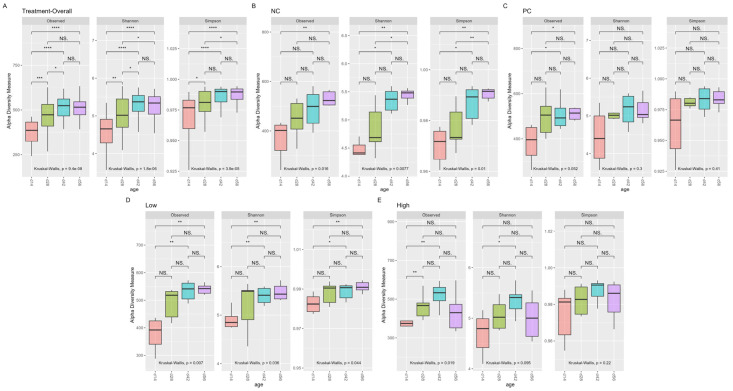
α-diversity indices (observed, Shannon, and Simpson) between timepoints grouped by treatment ((**A**): overall, (**B**): NC, (**C**): PC, (**D**): LOW, and (**E**): HIGH). Significant differences within each category were determined at *p* < 0.05 based on Kruskal–Wallis test. Significant differences between each factor were determined upon post hoc pairwise Wilcoxon rank-sum comparison at *p* < 0.05 (NS. ≥ 0.05, * < 0.05, ** ≤ 0.01, *** ≤ 0.001, **** ≤ 0.0001).

However, each collection timepoint tended to be significantly different from the others in most treatments (excluding PC treatment), as summarized in Table 3 and Figure 3, with the most pronounced differences often seen between d14 and d56 or the first and last four weeks of the study.

### 3.3. Βeta Diversity

**Figure 4:**
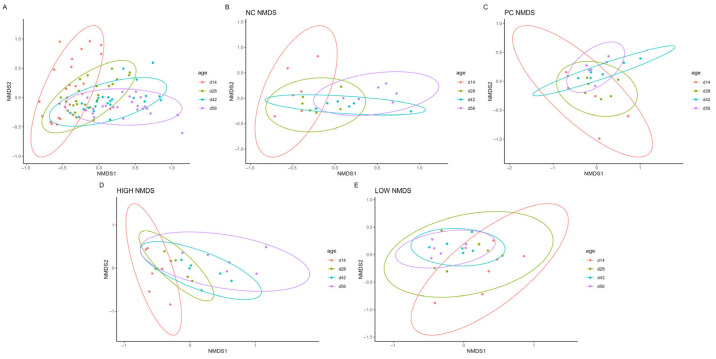
β-diversity non-metric multidimensional scaling (NMDS) between samples grouped temporally by treatments ((**A**): overall, (**B**): NC, (**C**): PC, (**D**): LOW, (**E**): HIGH).

Ordination of sample β-diversity was performed using non-metric multidimensional scaling (NMDS). Similarly to trends observed in α-diversity indices, the Bray–Curtis dissimilarity according to PERMANOVA/ANOSIM between ordinated treatments was generally not significant, except when considered as a whole (PERMANOVA: R^2^ = 0.174, *p* = 0.001; ANOSIM: R = 0.299, *p* = 0.0001) and on d56 (PERMANOVA: R^2^ = 0.196, *p* = 0.02; ANOSIM: R = 0.158, *p* = 0.15; Dispersion *p* = 0.015). However, distinct clusters formed between ordinated age groups when treatments were considered the predictor variable. Notably, the result for the HIGH treatment closely resembled that observed in the overall ordination (Figure 4, Table 4).

### 3.4. ASV Relative Abundance

**Figure 5:**
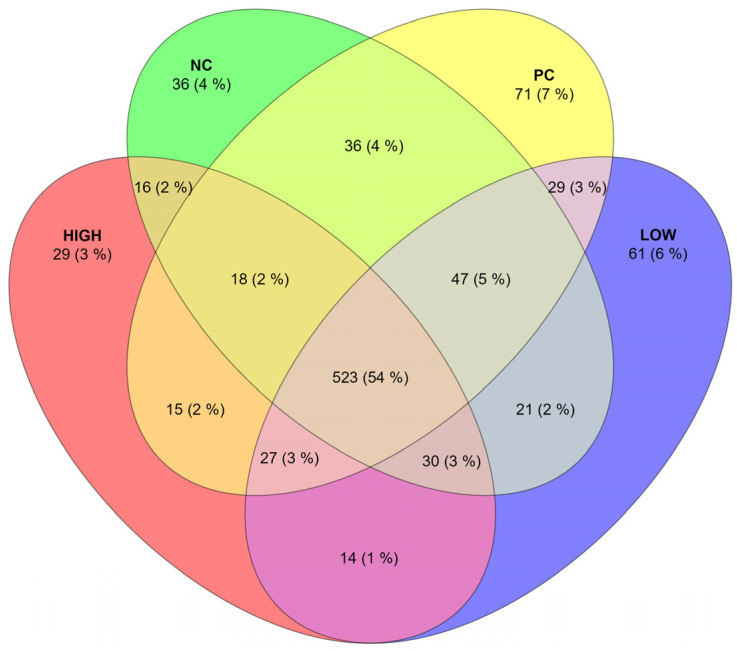
Composition of shared ASVs, between four treatment groups, present in 25% or more samples.

Of the 1571 ASVs assigned in 96 samples, 973 unique ASVs were found in 25% or more samples per treatment group. The count and percentage of the shared ASVs between all four treatment groups are listed in Figure 5.

Of the 523 shared ASVs between all treatment groups, 29.06% were not identified on the genus level (91.28% of which belonged to the Clostridia class, 5.8% to Bacilli, 0.78% to Gammaproteobacteria, 0.97% to Coriobacteria, and 1.16% to Bacteroidia) followed by *Mediterraneibacter* (14.72%) and *Blautia* (4.40%).

**Figure 6:**
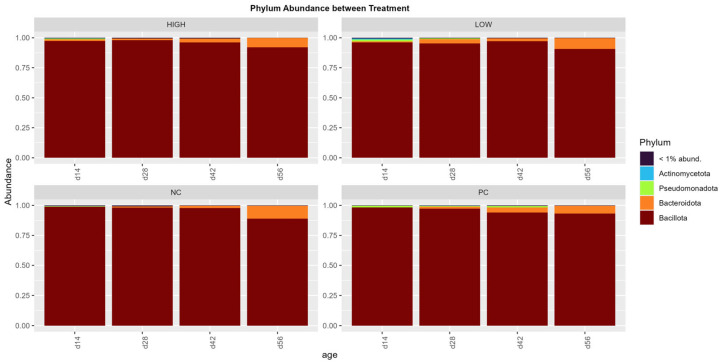
Amplicon sequence variant (ASV) abundance, pooled and grouped by treatments with collection timepoints/ages as factors (n = 6 per factor), on the phylum level.

**Figure 7:**
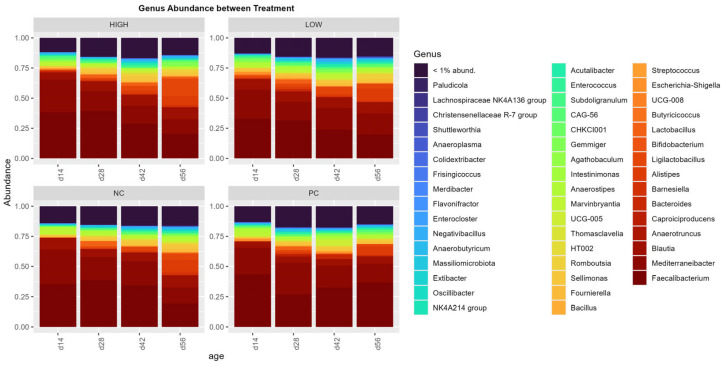
Amplicon sequence variant (ASV) abundance, pooled and grouped by treatments with collection timepoints/ages as factors (n = 6 per factor), on the genus level.

The phylum and genus abundances among the treatments are shown in Figure 6 and Figure 7, respectively. Overwhelmingly, Bacillota dominated the phylum abundance across all treatments (95.57%), followed by Bacteroidota (5.06%), Pseudomonadota (2.59%), and Actinomycetota (1.46%), with the Bacillota:Bacteroidota ratio being 18.87 on average (*p* < 0.0001).

## 4. Discussion

### 4.1. Bacterial Community Diversity

The use of prebiotics, probiotics, or their combination has been a popular choice for poultry producers to improve their flocks and lessen reliance on antibiotic treatments against diseases often faced by the poultry industry [52,53]. Such manners of usage have been well established and studied based on various parameters, including performance and overall bird health [16,54,55,56]. In contrast, much less has been examined in the context of BCO lameness—which is postulated to be related to gastrointestinal origins in nature [24,57]—and its associated etiology and pathogenesis. To date, there has been a general lack of studies investigating the various microbiomes associated with BCO lameness experiments [42,58,59], with the majority of research in the field focusing on the investigation of dietary treatments and vaccinations that may contribute to a reduction in disease prevalence. In particular, most published studies in this topic utilized populations of experimental animals reared under the mechanical BCO induction model described in Wideman et al. (2012) [32]. Despite its high effectiveness, this model is highly aggressive and potentially decreases translatability to a practical setting.

Previously, we investigated the use of a similar probiotic *E. faecium* spray on day-of-hatch broiler chicks and the associated BCO pathophysiological outcomes in an isolated, bacterially challenged environment through drinking water, with encouraging results regarding clinical lameness reduction [44]. While out of the research scope at the time, we hypothesized that early exposure to the probiotic *E. faecium* strain via spraying contributed to the attenuation of colonization—and thus subsequent negative effects—of the *Staphylococcus aureus* in the drinking water challenge that followed. At the same time, the use of lactic acid bacteria (LAB) has been documented to support gastrointestinal health through the strengthening of immune function modulation and the strengthening of epithelial tight junction proteins [60,61,62], both of which are influential factors in BCO etiology and pathology. Leveraging these findings, a focus on the gastrointestinal microbial communities, characterized by those belonging to the broiler caecum, may provide additional insights into disease etiology and the potential treatment mode of action. At the same time, usage of effective novel BCO induction models—such as the aerosol transmission model [31]—can potentially further augment research findings under more practical settings.

Similarly to other studies conducted previously using the same induction model [29,33,63], the cumulative clinical lameness incidence observed in this study closely resembled that of the all wire-flooring model, with seeder positive control (PC) pens reaching 77%. While somewhat lower compared to that found in several studies conducted in recent years, the cumulative clinical lameness incidence in negative control (NC) pens followed at 49%, being significantly different to that of treated pens (T3–T5/LOW and HIGH; *p* < 0.005), indicating the effectiveness of the treatment application. While undetermined, several uncontrollable factors may have contributed to this observed decreased rate in clinical lameness incidence in the NC treatment, including variations in flock susceptibility between hatches, as well as seasonality-based conditions. Specifically, while the health and performance of the experimental chicks were carefully monitored upon procurement in each experiment to maintain phenotypical uniformity, innate variations between different hatches—such as hatching conditions or hen age—and their implications for long-term health outcomes in a high-stress research environment were ultimately unavoidable [64,65]. Additionally, seasonality-based factors, such as temperature stress [66] and ventilation, may have also contributed to this difference. A closer investigation of these factors regarding BCO disease outcome is warranted, though such efforts to closely control finer details related to chick sources, the scheduling of facility space, and inadvertent weather conditions may present as significant hurdles to consider.

As shown, α-diversity indices grouped by each specific collection date (d14, 28, 42, or 56) showed no significant differences between treatments, indicating similar degrees of diversity, richness, and evenness within each group. The opposite was observed temporally, with increasing community richness and evenness at greater ages compared to earlier stages of life. Similar trends could be observed in measurements of β-diversity, with significant differences identified as the birds aged. Together, these findings are consistent with those of other studies characterizing the broiler cecal microbiome [67,68,69], where the temporal impact of age and development have significant influence on the gastrointestinal microbial community, with species diversity naturally increasing from hatch and stabilizing as the birds age. Interestingly, both richness and evenness measures seemed to have decreased between the last two collection timepoints on d42 and d56, particularly in the HIGH treatment—though no significant difference between treatments was observed. This coincides with the general period during which the clinical lameness incidence rate in BCO studies often peaks [17,29,43]—including this study. A decrease in microbial community richness and evenness typically implies a reduction in species diversity, which may be influenced by both developmental and environmental stress. In the context of this study, the rapid and persistent weight gain of experimental broilers, coupled with the aerosolized transmission of shed etiological agents in the lameness induction model, can be considered highly stressful to experimental birds in general, which may partially provide an answer to this observed phenomenon in our experiment—though this does not adequately address the non-significant decrease in richness/evenness measures observed solely in the HIGH group. Another possible explanation may be the low number of biological replicates, which will be discussed in a later section. Regardless, further detailed in vivo and in vitro [27] investigations are needed for a more complete picture regarding this matter.

In terms of species abundance, an immediately observable trend was the overwhelming domination of the phylum Bacillota (Firmicutes) in all populations across different ages, followed by an apparent minor increase in the abundance of Bacteroidota during later stages of life. This starkly contrasts the conventional broiler gastrointestinal microbiome, where the Bacillota:Bacteroidota ratio is typically much lower and the presence of other phyla is significantly more apparent [45,70], possibly signifying the abnormal development of the cecal microbial microbiome in all treatment populations. Remarkably, while the commonly involved experimental models were different from that used in this study, such a drastic difference between cecal Bacillota and Bacteroidota composition has been previously documented in nutrition studies involving oligosaccharide prebiotics in which broilers were reared under both normal and suboptimal conditions [71,72,73]. Even though a higher Bacillota to Bacteroidota ratio has been implicated as a potential biomarker for the diagnosis of obesity in humans [74], this remains under debate, which may be due to technical differences in sampling and analyses between studies [75] as well as the potential inherent biological differences between individuals that cannot be universally characterized [76]. Similarly, this characterization has yet to be definitively identified in broilers, with various studies producing contradictory results regarding broiler weight outcomes [77,78,79]. Regardless, several factors unique to this study may provide potential answers to the observed phenomenon. The intense photoperiod schedule employed in the aerosol transmission model (23 L:1 D for 56 days) was designed to prolong broiler activity, feed consumption, and rapid weight increase associated with the triggering of tibial and femoral microfractures and resultant lameness [24,31]. This may be a potential factor contributing to the observed Bacillota:Bacteroidota ratio. Several studies in the current literature have offered early insights into the potential impact of the photoperiod on broiler cecal communities, though findings remain scant and inconsistent [80,81]. In the context of this study, prolonged broiler activity periods, coupled with the stress of persistent infection introduced aerobically by the induction model, may have resulted in impaired gut modulation functions across the board [81,82] and the eventual overabundance of Bacillota, as similar cases of dysbiosis have been linked to these factors in a mouse model with hypertension [83].

### 4.2. Limitations, Considerations, and Future Suggestions

A significant limitation of this study was the low number of sampled animals per collection timepoint. Current broiler BCO research models—particularly those investigating population dynamics—require a high number of birds per experiment for consistent disease spreading based on standard industrial stocking density rates, as well as accurate cumulative lameness incidence and mortality analyses. At four collection timepoints and three biological replicates each, which is a common number for statistical investigations, the eventual loss of birds per treatment pen from sample collection will have been 24% (out of the predetermined 50 birds per pen, as outlined in our experimental design). At the same time, the lack of proper alternatives for the collection of samples and/or tissues, such as cecal content, that necessitate sacrifice of the animal further adds to this dilemma. As such, the number of clinically normal birds chosen for terminal sampling, especially for temporal comparisons, must be carefully considered to avoid impacting the remaining population, as these birds can neither be considered normal mortality nor lameness cases. While not possible for this study, future research examining the same parameters should explore increasing this number of sampled animals to two per pen (16% at four collection timepoints), as well as reducing sampling dates, if possible.

**Figure 8:**
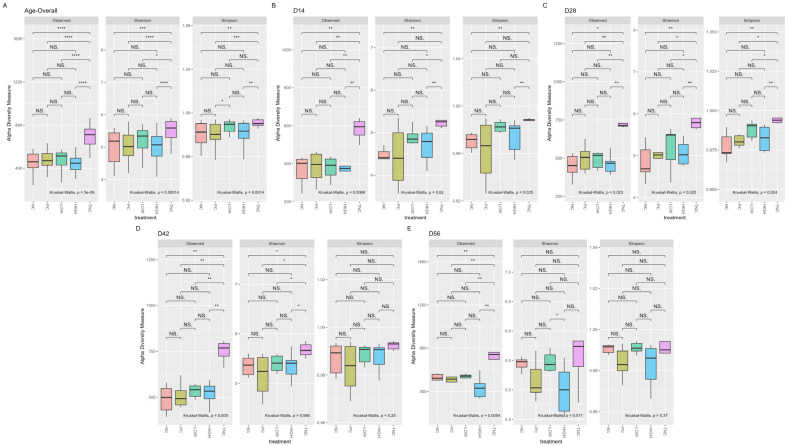
α-diversity indices (observed, Shannon, Simpson) between samples grouped by collection timepoints/ages ((**A**): overall, (**B**): d14; (**C**): 28; (**D**): 42; (**E**): 56) and treatments (including “True” NC, TNC) as factors. Significant differences within each category were determined at *p* < 0.05 based on Kruskal–Wallis test. Significant difference between each factor determined upon post hoc pairwise Wilcoxon rank-sum comparison at *p* < 0.05 (NS. ≥ 0.05, * < 0.05, ** ≤ 0.01, *** ≤ 0.001, **** ≤ 0.0001).

**Figure 9:**
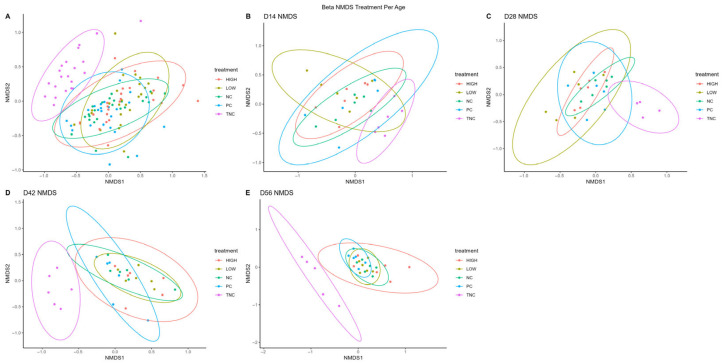
β-diversity non-metric multidimensional scaling (NMDS) between samples, grouped by collection timepoints/ages ((**A**): overall, (**B**): d14, (**C**): 28, (**D**): 42, (**E**): 56) and treatments (including “True” NC, TNC) as factors.

Due to space and sourcing issues at the time the original experiment was planned, the housing environment was strictly isolated to the same unit at the research site (House 365 W, University of Arkansas Poultry Research Facility), which has traditionally been used as our group’s research barn over the years. While the barn undergoes extensive disinfection and all treatment pens are newly refabricated with fresh wood shavings for each experiment, the mechanisms behind the aerosol transmission model did not allow for a “true” negative control group for direct comparison with an uninfluenced population free from disseminated etiological agents in the same barn. Following suggestions made by Mandal et al. (2020) [42] regarding the lack of a “true” negative control with completely isolated birds reared under a non-stress environment in BCO lameness research, preliminary comparisons in the cecal microbiome were attempted with previously sequenced data from an isolated NC population (Figure 8 and Figure 9).

While clear significant differences are apparent in both α- and β-diversity indices, these results should only be considered for contextual purposes due to the major differences in our experimental design (in terms of chick source, diet phase, and diet composition). Nonetheless, this echoes the need for a consideration of an isolated experimental population in broiler BCO lameness research, where the main experimental housing invariably becomes a “hot” zone unsuitable for unbiased baseline parameters regardless of the induction model used. A simple and direct possible solution is the inclusion of a sub-population reared entirely in a physically isolated housing unit, which should come from the same hatch and have identical (or very similar) environmental conditions to the main research unit—including light schedule, thermoneutral temperature ranges, ventilation, and number of replicate pens.

Evidently, although the results presented here are inconclusive as to whether the administration of the probiotic treatments directly correlated with the reduction in clinical lameness rates observed in terms of the influence of the cecal community in experimental broilers, the distinctively tight clustering in the β-diversity ordination of the HIGH treatment (combination of sprayed and diet probiotics) observed in Figure 5 warrants further examination. Therefore, future research investigating the BCO induction mode of action in the aerosol transmission model is needed, with additional investigations of relevant extraintestinal sites such as those described in Mandal et al. (2020) [42] and the upper respiratory tract. Furthermore, preliminary work measuring the expression of genes regulating both upper respiratory and gastrointestinal tissue integrity, such as the CLDN family, has shown increases in selected prebiotic/probiotic treatments linked to reduced clinical lameness under the same induction model. Considering that there may be multiple portals of entry through which broilers may acquire BCO-causative bacteria [24], there are reasonable grounds to believe that effective preventive treatments may contribute to the modulation of extraintestinal immunomodulatory functions and microbiomes (such as the respiratory tract), thus affecting bacterial translocation events and/or immunological defense against etiological agents in a multi-pronged fashion, ultimately affecting clinical BCO lameness rates under this model.

## 5. Conclusions

This study evaluated the cecal microbiota composition in broilers reared in treatment groups involving a day-of-hatch probiotic *E. faecium* spray and as well as in groups containing a combination of this spray with probiotic *B. amyloliquefaciens*/*B. subtilis* inclusion in diet under high-stress conditions, using the aerosol transmission BCO induction model. While no significant effects were found between treatments within each collection timepoint, the temporal diversity indices were significantly different between each other. The preliminary findings presented in this study are—to our knowledge—among the first to have resulted from an investigation of the gastrointestinal microbiome, characterized by cecal microbial communities, in experimental broilers reared under the aerosol transmission induction model. Future research should focus on additional extraintestinal sites and parameters of investigation, as well as the use of an isolated “true” negative control population for relevant biological comparisons.

## Figures and Tables

**Table 1:** Selected experimental populations for study evaluation.

Population	Treatment	Pen Type
Positive Control—PC	None	Wire
Negative Control—NC	None	Litter
Probiotic Spray—LOW	*E. faecium* 669 @ 2 × 10^9^ CFU/bird on d0	Litter
Probiotic Spray + Diet—HIGH	*E. faecium* 669 @ 2 × 10^9^ CFU/bird on d0 + *B. amyloliquefaciens* 516/*B. subtilis* 597/*B. subtilis* 600 @ 492.1 mg/kg feed for 56 d	Litter

**Table 2:** Statistical comparison of cumulative lameness incidence between selected experimental treatment groups on d56.

*p*-Value	NC	LOW	HIGH
PC ^†^	0.0036 *	0.0037 *	0.0086 *
NC		<0.0004 *	<0.0001 *
LOW			0.0133 *

Note: An asterisk (*) denotes a statistically significant difference (*p* < 0.05). Treatment groups are defined as follows: PC = positive control, NC = negative control, LOW = Probiotic *E. faecium* spray @ 2 × 10^9^ CFU/bird on d0, and HIGH = Probiotic *E. faecium* spray @ 2 × 10^9^ CFU/bird on d0 + Triple-strain *Bacillus*-based probiotic feed additive the in diet from d0–56. ^†^ The statistical analysis for PC treatment is presented for informational purposes only due to the replication of different treatment pens.

**Table 3:** Occurrences of significant differences in α-diversity indices, grouped by treatments.

Treatment	Observed	Shannon	Simpson
NC	0.016	0.0077	0.01
PC	NS	NS	NS
LOW	0.007	0.036	0.044
HIGH	0.019	NS	NS

**Table 4:** Occurrences of significant differences identified in measurements of β-diversity, grouped by treatment as the predictor.

Treatment	PERMANOVA (R^2^; *p*)	ANOSIM (R; *p*)	Dispersion (*p*)
Overall	0.174; 0.001	0.299; 0.0001	0.026
NC	0.302; 0.001	0.383; 0.0001	NS
PC	0.218; 0.008	0.202; 0.002	NS
LOW	0.250; 0.001	0.323; 0.0002	0.004
HIGH	0.291; 0.001	0.347; 0.0002	NS

## Data Availability

Data can be made available from authors upon reasonable request.

## Abbreviations

The following abbreviations are used in this manuscript:

BCO	Bacterial Chondronecrosis with Osteomyelitis
PC	Positive Control
NC	Negative Control
GIT	Gastrointestinal Tract
